# Streptozotocin-induced diabetes prolongs twitch duration without affecting the energetics of isolated ventricular trabeculae

**DOI:** 10.1186/1475-2840-13-79

**Published:** 2014-04-15

**Authors:** June-Chiew Han, Kenneth Tran, Poul MF Nielsen, Andrew J Taberner, Denis S Loiselle

**Affiliations:** 1 Auckland Bioengineering Institute, The University of Auckland, Auckland, New Zealand; 2 Department of Engineering Science, The University of Auckland, Auckland, New Zealand; 3 Department of Physiology, The University of Auckland, Auckland, New Zealand

**Keywords:** STZ-induced diabetes, Cardiac work, Cardiac heat production, Cardiac efficiency, Cardiac twitch duration

## Abstract

**Background:**

Diabetes induces numerous electrical, ionic and biochemical defects in the heart. A general feature of diabetic myocardium is its low rate of activity, commonly characterised by prolonged twitch duration. This diabetes-induced mechanical change, however, seems to have no effect on contractile performance (i.e., force production) at the tissue level. Hence, we hypothesise that diabetes has no effect on either myocardial work output or heat production and, consequently, the dependence of myocardial efficiency on afterload of diabetic tissue is the same as that of healthy tissue.

**Methods:**

We used isolated left ventricular trabeculae (streptozotocin-induced diabetes versus control) as our experimental tissue preparations. We measured a number of indices of mechanical (stress production, twitch duration, extent of shortening, shortening velocity, shortening power, stiffness, and work output) and energetic (heat production, change of enthalpy, and efficiency) performance. We calculated efficiency as the ratio of work output to change of enthalpy (the sum of work and heat).

**Results:**

Consistent with literature results, we showed that peak twitch stress of diabetic tissue was normal despite suffering prolonged duration. We report, for the first time, the effect of diabetes on mechanoenergetic performance. We found that the indices of performance listed above were unaffected by diabetes. Hence, since neither work output nor change of enthalpy was affected, the efficiency-afterload relation of diabetic tissue was unaffected, as hypothesised.

**Conclusions:**

Diabetes prolongs twitch duration without having an effect on work output or heat production, and hence efficiency, of isolated ventricular trabeculae. Collectively, our results, arising from isolated trabeculae, reconcile the discrepancy between the mechanical performance of the whole heart and its tissues.

## Background

A plethora of electrical, ionic and biochemical phenotypes characterises the myocardium of the STZ-induced diabetic rat. Prolongation of action potential duration [1-6] reflects reduction of steady-state [7,8] and transient outward K^+^ currents [4,5,7-10], including the delayed rectifier [1], all of which changes are attributable to down-regulation of K^+^ channel gene expression [10]. These changes result in slowing the rate of diastolic depolarization [1] and, in consequence, spontaneous rate [1]. These sarcolemmal alterations anticipate comparable changes of sarcoplasmic reticular behaviour: prolongation of the Ca^2+^ transient [6,11-13], and subsequent decrease of L-type Ca^2+^ current [1,7]. Contiguous with these electrical and ionic changes are reductions in activity of their associated membrane-bound ATPases: the sarcolemmal Na^+^-K^+^-ATPase [14-16] and the sarcoplasmic reticular Ca^2+^-ATPase [17-22]. Comparable slowing of down-stream mechanical events reflects diminution of the myofibrillar-ATPase [23-28], with a shift of myosin heavy chain (MHC) isoenzyme pattern towards the (slow) β-isoform [20,24,26,29,30].

The constellation of electrical, ionic, and biochemical responses outlined above comprises the ‘diabetic signature’ and is compatible with the well-known diabetes-induced prolongation of the twitch, consistently shown at all levels of the streptozotocin (STZ)-induced diabetic myocardium: hearts [31-33], papillary muscles [2,3,28,30,34-37], ventricular trabeculae [6,12], and single myocytes [13,31,38,39]. However, an inconsistency exists between the data arising from the diabetic heart and that arising from its isolated tissues. The peak left-ventricular (LV) systolic pressure development is reduced in diabetes [27,40-45], but both the stress (force per cross-sectional area) produced by isolated papillary muscles [2,3,28,30,34-37], and the peak shortening of single myocytes [13,46-49], remain unaffected.

**Figure 1:**
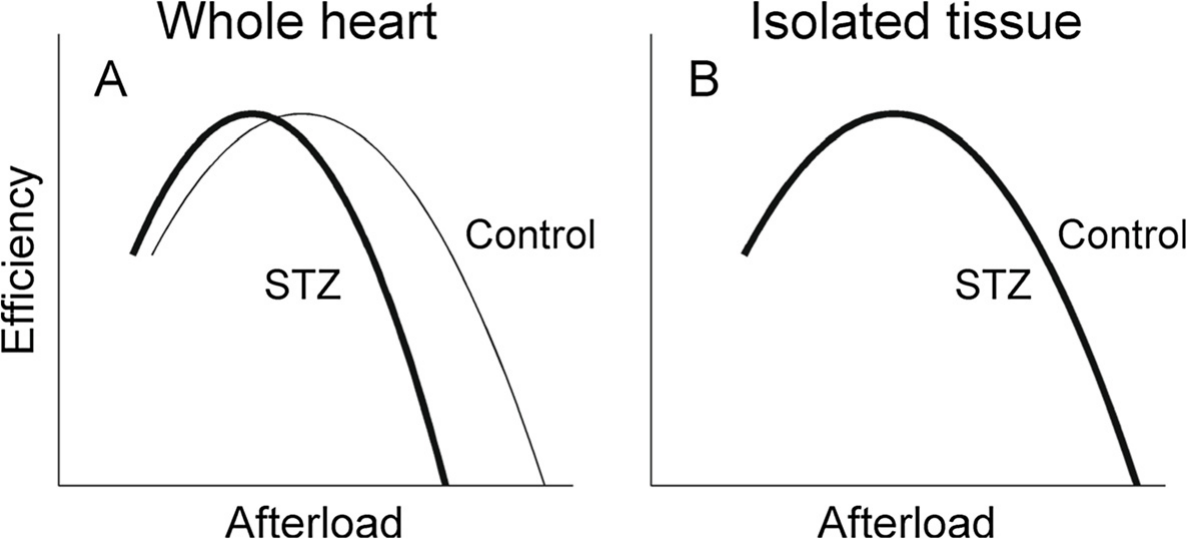
**Qualitative comparison between whole heart data and hypothesised tissue data. (A)** observed dependence of myocardial efficiency on afterload for the whole heart (STZ-diabetes versus Control). **(B)** putative efficiency-afterload relation of tissue isolated from the STZ-diabetic heart - the same as that isolated from the Control heart.

We have recently demonstrated [50] that the STZ-diabetic whole heart *in vitro* fails to generate as high a pressure as healthy hearts when confronted by high afterloads (Figure 1A). That is, diabetes limits the ability of the heart to pump at high afterloads, which consequently results in a left-shift of the myocardial efficiency-afterload relation, but without affecting the peak value of efficiency. This limitation we presume to arise as a consequence of insufficient LV diastolic filling due to diabetes-induced prolongation of twitch duration and consequent abbreviation of diastole.

Given the above considerations, we hypothesise that efficiency-afterload relations will be no different between the tissues isolated from diabetic and control hearts (Figure 1B). To test this hypothesis, we utilised isolated left ventricular trabeculae. The use of isolated cardiac tissues eliminates the intrinsic complexity of the ventricle and obviates the effect of insufficient diastolic filling.

## Methods

### Ethical approval

All the procedures of handling and use of animals were approved by the University of Auckland Animal Ethics Committee (Approval R925).

### Animal preparation

Diabetes was induced by a single tail-vein injection of streptozotocin (STZ, 55 mg kg^-1^) administered to male Sprague–Dawley rats (6 weeks - 7 weeks old, 250 g - 300 g). Age- and mass-matched control rats received intravenous injections of equivalent volumes of saline. Rats in each group were housed (in pairs) in a cage in a room (20°C - 22°C) on a 12-hr light/dark cycle. They had *ad lib* access to food (standard rat chow) and tap water. Induction of diabetes in the STZ-treated rats was confirmed by their elevated levels of blood glucose (>20 mM), measured daily using a glucometer for a week post-injection and thence once per week for 7 weeks - 8 weeks.

### Preparation of trabeculae

On the day of an experiment (7 weeks - 8 weeks post-injection), a rat was deeply anaesthetised with isoflurane (<5% in O_2_) and, following measurement of its body mass, injected with heparin (1000 IU kg^-1^) prior to killing by cervical dislocation. The excised heart was plunged into chilled Tyrode solution, and the aorta immediately cannulated for Langendorff perfusion with Tyrode solution (in mM: 130 NaCl, 6 KCl, 1 MgCl_2_, 0.5 NaH_2_PO_4_, 1.5 CaCl_2_, 10 Hepes, 10 glucose; pH adjusted to 7.4 by addition of Tris) vigorously gassed with 100% O_2_ at room temperature. Once the coronary vasculature was clear of blood, the heart was mounted on a working-heart rig and subjected to a series of pressure-volume work-loops at a range of afterloads, while measuring its rate of oxygen uptake. At the end of this experiment (which typically lasted four hours), the details of which can be found in [50], the heart was removed from the rig and again Langendorff-perfused with dissection solution (Tyrode solution with Ca^2+^ reduced to 0.3 mM and supplemented with 20 mM 2,3-butanedione monoxime). The left ventricle (LV) was opened and trabeculae (typically located around the papillary muscles) were dissected. A geometrically uniform LV trabecula was then mounted onto a pair of hooks in our work-loop calorimeter [51] and superfused (at a rate of 0.5 μL s^-1^ - 0.7 μL s^-1^) with Tyrode solution. During an equilibration period (at least one hour), the trabecula, while electrically stimulated to contract at 3 Hz, was gradually stretched to optimal length (*L*_*o*_; the length that maximises developed force). In total, 15 Control trabeculae and 12 STZ trabeculae, obtained respectively from 13 Control hearts and 11 STZ hearts, were used. The trabeculae did not differ between groups (Control and STZ) in either average diameters (227 μm ± 17 μm and 195 μm ± 17 μm) or average lengths (2.78 mm ± 0.18 mm and 2.58 mm ± 0.33 mm).

### Experimental protocol

During an experiment, the entire calorimeter system was enclosed by a light-proof and thermally-insulated lid, with the temperature within the enclosure maintained at 32°C. Each experiment commenced with a trabecula contracting isometrically at *L*_*o*_ while paced at 3 Hz (chosen because this frequency approximates the intrinsic rate of the isolated rat heart at 32°C [50], and avoids the increase of diastolic force that accompanies higher rates) until achieving steady states of force and rate of heat production. The trabecula was then required to perform a train of workloop contractions (bracketed by isometric contractions) at a range of afterloads [51,52], in decreasing order. Note that the force-length work-loops were designed to mimic the pressure-volume loop of the heart. This procedure was repeated until the afterload was in the vicinity of zero developed force. Upon completion of this ‘work-loop protocol’, the trabecula was required to undergo an ‘isometric protocol’, during which it was subjected to isometric contractions at progressively diminishing muscle lengths, commencing at *L*_*o*_ and proceeding to the length which produced near zero developed force. Lastly, the trabecula was required to complete a ‘length-perturbation protocol’ for measurement of dynamic stiffness [53].

### Measurement of dynamic modulus

In order to interrogate crossbridge status, muscle dynamic stiffness was computed, as described previously [53]. In this protocol, muscle length was sinusoidally perturbed at 100 Hz at a constant amplitude of 0.001 L_o_. Muscle length perturbation (∆*L*) and the resulting oscillation of twitch force (∆*F*) were recorded throughout the entire time courses of twitches (3 Hz and 6 Hz stimulus frequencies). A sliding window of 7 ms width was advanced in steps of 1 ms throughout each steady-state length and twitch force profile. Within this window, ∆*L* and ∆*F* traces were fitted by sinusoidal curves: *ΔX* = *A*_*X*_ ⋅ sin(2*πft*) + *B*_*X*_ ⋅ cos(2*πft*) + *C*_*X*_ ⋅ *t* + *D*_*X*_, where *X* represents either *L* or *F*, *f* is perturbation frequency (100 Hz), *t* is time, and *A*, *B*, *C* and *D* were obtained through least-squares optimisation. Dynamic stiffness was calculated as $\sqrt{A_{F}^{2}+B_{F}^{2}}/\sqrt{A_{L}^{2}+B_{L}^{2}}$, and mean *F* as *C*_*F*_ ⋅ *t*_*c*_ + *D*_*F*_ , where $t_{c}$ is the time at the centre of the window. Dynamic modulus was calculated as the product of dynamic stiffness and *L*_*m*_/*A*_*m*_, where *L*_*m*_ and *A*_*m*_ are muscle length and cross-sectional area, respectively.

### Corrections for thermal artefacts

Three thermal artefacts sully the measurement of muscle heat production. One arises from the slow exponential drift of temperature with time inside the insulated enclosure when experiments are performed at temperatures above that of the room. Correction for this behaviour was effected by subtracting an exponential function fitted to the heat record obtained with the trabecula in the measurement chamber but in the quiescent state. The contributions from the remaining two sources were determined at the end of the experiment. During a train of work-loop contractions, the downstream hook and glass connecting rod are required to move upstream, repeatedly, to allow the muscle to shorten. This cyclical movement generates a heat artefact, thereby requiring correction. The magnitude of this source, typically <10% of the peak total measured signal (when the muscle performed isometric contractions at *L*_*o*_), was quantified by mimicking the train of workloop contractions with the stimulator off and the muscle quiescent. Stimulus heat was measured in the absence of the trabecula. The magnitude of the stimulus heat (3 Hz, and typically 3 V and 3 ms stimulus pulses) was commonly about 10% of the peak total measured signal.

### Definitions and normalisations

Muscle force (N) was converted to stress (kPa) by dividing by muscle cross-sectional area. Muscle length was expressed relative to optimal muscle length (*L*/*L*_o_). A stress-length workloop has four distinct phases [51]: isometric contraction, isotonic shortening, isometric relaxation and isotonic re-lengthening. For a workloop twitch, ‘afterload’ was defined as the user-selected stress at which the muscle transitioned from the isometric phase to isotonic shortening. ‘Relative afterload’ (*S*/*S*_o_) is the ratio of the afterload (*S*) to the peak isometric total (active plus passive) stress (*S*_o_). ‘Active afterload’ was defined as the difference between afterload (*S*) and passive stress at *L*_*o*_, and hence ‘relative active afterload’ was defined as the ratio of ‘active afterload’ to peak isometric active stress (at *L*_*o*_). Muscle ‘shortening’ was defined as the difference between *L*_*o*_ and the end-systolic length reached during a workloop contraction (i.e., at the point of transitioning between isotonic shortening and isometric relaxation) and was expressed as a percentage of *L*_*o*_. Maximum velocity of muscle shortening for each workloop (*V*_*s*_) was computed from the length-time trace during the isotonic shortening phase. Its value was normalised to muscle length and was thus expressed in units of s^-1^. Power of shortening (*P*_*s*_) is the product of *V*_*s*_ and ‘active afterload’. External mechanical work is the area of the workloop (calculated by integrating stress as a function of *L*/*L*_o_ over the entire period of the twitch) and was expressed in units of kJ m^-3^. Change of enthalpy (kJ m^-3^) is the sum of work and heat. Mechanical efficiency is the ratio of work to change of enthalpy and was thus expressed as a percentage. For calculation of crossbridge efficiency, the denominator of the foregoing ratio was reduced by subtraction of the heat of activation of work-loop contractions (extrapolated as the intercept of the heat-*S*/*S*_o_ relation). The duration of the twitch (expressed in *ms*) was quantified at 5% and 50% of the peak stress [54]. For an isometric twitch, the area under the twitch profile is defined as the stress-time integral (*STI*) and has units of kPa s. The maximum rates of rise and fall (*±dS/dt*; MPa s^-1^) of twitch stress were computed from the ascending and descending limbs of the twitch, respectively.

### Curve fitting

For the data obtained from the ‘isometric protocol’, total stress and passive stress were plotted against *L*/*L*_o_ and fitted using 3^rd^-order polynomials. Twitch duration, and ±*dS/dt*, were plotted against active stress and fitted using 1^st^-order polynomials. *STI* was plotted against active stress and fitted using 2^nd^-order polynomials. Heat was plotted against both active stress and *STI*. These relations were fitted using 2^nd^-order and 1^st^-order polynomials, respectively. For the ‘work-loop protocol’, shortening was plotted against ‘relative active afterload’ and fitted using a 2^nd^-order polynomial. Velocity of shortening ($V_{S}$) was plotted against relative active afterload (*S*_*A*_/*S*_*o*_) and fitted using the Hill hyperbolic velocity-load function: *V*_*S*_ = *b*(*c* − *S*_*A*_/*S*_*o*_)/(*a* − *S*_*A*_/*S*_*o*_), where *a*, *b* and *c* were obtained through least-squares optimisation. Note that we did not constrain *V*_*S*_ to pass through *S*_*A*_/*S*_*o*_ = 1, given that *V*_*S*_ − *S*_*A*_/*S*_*o*_ data are poorly described by the Hill function when *S*_*A*_/*S*_*o*_ > 0.8 [55]. The relation between shortening power (*P*_*S*_) and *S*_*A*_/*S*_*o*_ was derived from the *V*_*S*_ − *S*_*A*_/*S*_*o*_ relation. Heat and change of enthalpy were plotted against relative afterload *S*/*S*_*o*_ and fitted using 1^st^-order and 2^nd^-order polynomials, respectively. Work and both mechanical and crossbridge efficiencies were plotted against *S*/*S*_*o*_ and fitted using 3^rd^-order polynomials constrained to pass through (0, 0) and (0, 1).

### Statistical analyses

The regression lines (each obtained from a single trabecula) were averaged within groups using either the ‘random coefficient’ model within *Proc Mixed* for 1^st^-order and 2^nd^-order polynomials, or *Proc Nlmixed* for 3^rd^-order polynomials, of the SAS software package (SAS Institute Inc., Cary, USA). The ‘random coefficient’ model treats the regression coefficients arising from measurements made in individual trabeculae as a random sample from a multivariate normal population of possible coefficients [56].

Parameters of interest (peak values of shortening, shortening velocity, shortening power, work and efficiency), estimated from the appropriate regression lines, were averaged and compared between the Control and STZ groups. The data arising from isometric contractions (i.e., peak values of twitch stress, twitch duration and twitch heat) were averaged and compared between the Control and STZ groups. Analysis of variance was performed for each of these variables, using the ‘generalised linear model’ of SAS, accounting for both the variability among trabeculae from different hearts and that between trabeculae within individual hearts. All values were expressed as mean ± standard error (SE). Statistical significance was declared when *p* < 0.05.

## Results

### Morphometric characteristics of the rats

Morphometric characteristics of the subset of hearts from rats [50] that yielded trabeculae of suitable dimensions are presented in Table 1. A single injection of streptozotocin (STZ) resulted in hyperglycaemia. Compared with the Control rats, the STZ-diabetic rats were smaller, as indicated by their lower average body masses and average shorter tibial lengths. STZ-treatment also resulted in LV hypertrophy, as evident by the increased average septal and LV wall thicknesses (relative to heart wet mass) of the STZ rats.

**Table 1:** General characteristics of control and STZ rats

**Parameter**	**Control ( *n *** **=13)**	**STZ ( *n *** **=11)**
Body mass (g)	506 ± 16	363 ± 15*
Tibial length (mm)	44.6 ± 0.6	41.9 ± 0.8*
Blood Glucose (mM)	6.9 ± 0.1	30.0 ± 0.6*
Heart wet mass (g)	1.42 ± 0.05	1.13 ± 0.04*
Heart dry mass (g)	0.27 ± 0.01	0.22 ± 0.01*
Heart wet mass/body mass (%)	0.28 ± 0.01	0.31 ± 0.01*
RV wall thickness (mm)	1.29 ± 0.05	1.18 ± 0.08
RV thickness/heart wet mass (mm g^-1^)	0.92 ± 0.04	1.07 ± 0.09
Septal wall thickness (mm)	3.17 ± 0.10	3.02 ± 0.13
Septal thickness/heart wet mass (mm g^-1^)	2.25 ± 0.07	2.72 ± 0.17*
LV wall thickness (mm)	3.82 ± 0.07	3.65 ± 0.12
LV thickness/heart wet mass (mm g^-1^)	2.72 ± 0.11	3.26 ± 0.12*

Values are means ± SE. *P < 0.05.

### Isometric contractions

**Figure 2:**
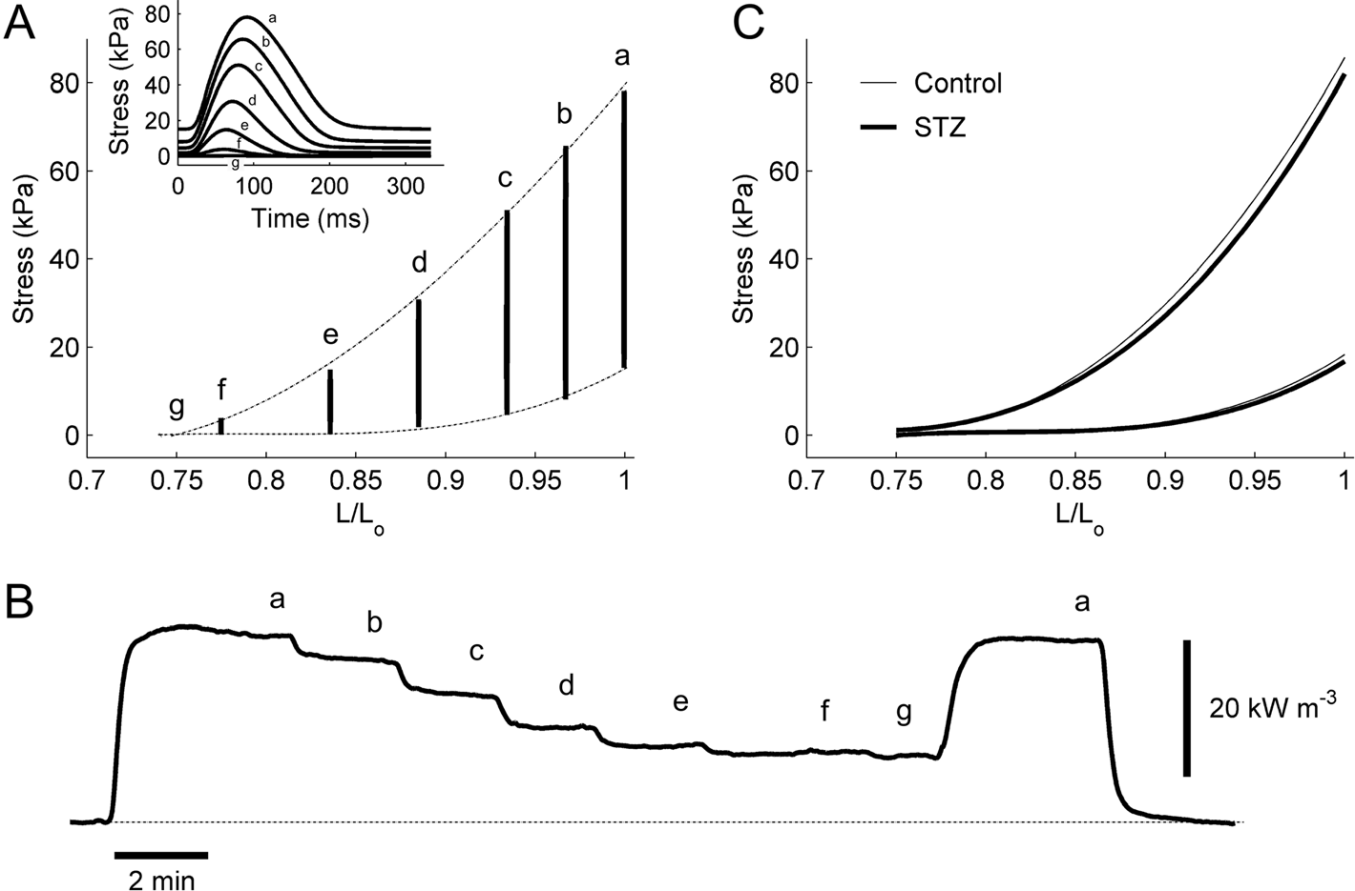
**Isometric stress-length relations and original record of rate of heat production at 3 Hz stimulation. (A)** Steady-state isometric stress-length relation of a representative trabecula. The upper and lower broken lines represent the fitted total and passive stress-length relations, respectively. The inset shows the individual twitch stress profiles (in order of decreasing muscle length, *a* to *g*). **(B)** Record of rate of heat production of a representative trabecula undergoing isometric contractions at progressively diminishing muscle lengths (*a* to *g*). **(C)** Average total (upper lines) and passive (lower lines) stress-length relations for the *n* = 15 Control (thin lines) and *n* = 12 STZ (thick lines) trabeculae.

Each trabecula (*n* = 15 Control and *n* = 12 STZ) was subjected to isometric contractions in decreasing order of muscle length (i.e., ‘isometric protocol’). Twitch stress at steady state was plotted against relative muscle length (*L/L*_*o*_) and quadratic regression lines used to fit separately to the total stress and passive stress (Figure 2A). The relations of the total stress-length and passive stress-length for all the trabeculae in each group were averaged (Figure 2C). Diabetes had no effect on these relations. The average peak stresses (total stress, active stress and passive stress), obtained at *L = L*_*o*_, did not differ between the Control (84.7 kPa ± 7.7 kPa, 66.3 kPa ± 6.9 kPa and 18.3 kPa ± 1.8 kPa) and the STZ trabeculae (81.1 kPa ± 8.0 kPa, 64.7 kPa ± 6.7 kPa and 16.4 kPa ± 2.8 kPa). The measured rate of heat production (Figure 2B) was converted to heat per twitch by dividing by stimulus frequency (3 Hz). Peak twitch heat did not differ between groups (11.2 kJ m^-3^ ± 1.0 kJ m^-3^ for Control and 11.0 kJ m^-3^ ± 1.0 kJ m^-3^ for STZ).

### Isometric twitch duration

**Figure 3:**
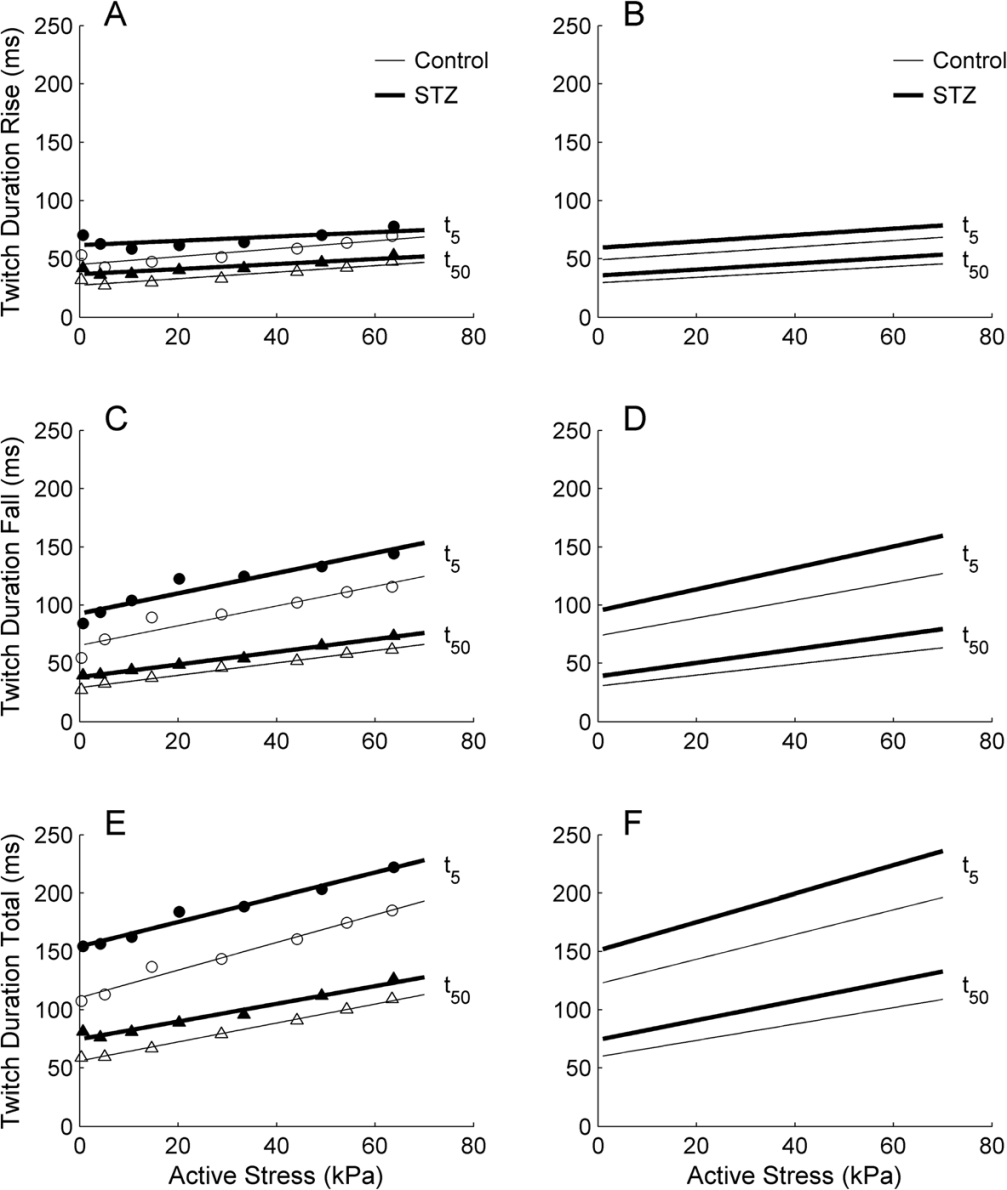
**Isometric twitch durations as functions of active stress at 3 Hz stimulation.** Time courses of the contraction phase **(A and B)** and relaxation phase **(C and D)** and their sum **(E and F)** of the isometric twitch, quantified at 5% (t_5_) and 50% (t_50_) of peak stress. Data from a representative trabecula from the Control group (open symbols) and from the STZ group (filled symbols) (left panels) and the average relations for *n* = 15 Control (thin lines) and *n* = 12 STZ (thick lines) trabeculae (right panels).

The duration of isometric twitch stress was quantified at 5% and 50% of peak stress, and was partitioned into contraction and relaxation phases. These were plotted against active stress (Figure 3). Diabetes prolonged the time courses of both contraction (Figure 3A and B) and relaxation (Figure 3C and D), i.e., the average duration-stress regression lines of the STZ trabeculae were higher than those of the Control trabeculae. The average peak values (obtained when *L = L*_*o*_) of twitch duration (contraction, relaxation and total) at 5% of peak stress were greater for the STZ trabeculae (79.2 ms ± 1.5 ms, 144.2 ms ± 2.9 ms and 223.5 ms ± 2.1 ms) than for the Control trabeculae (68.5 ms ± 1.8 ms, 120.3 ms ± 3.4 ms and 188.8 ms ± 4.5 ms). Similar results obtained at 50% of peak stress (STZ: 53.1 ms ± 1.2 ms, 73.8 ms ± 1.3 ms and 126.9 ms ± 1.7 ms; Control: 45.5 ms ± 1.3 ms, 60.3 ms ± 2.0 ms and 105.7 ms ± 3.0 ms).

### Rates of rise and fall of isometric twitch stress and stress-time integral

**Figure 4:**
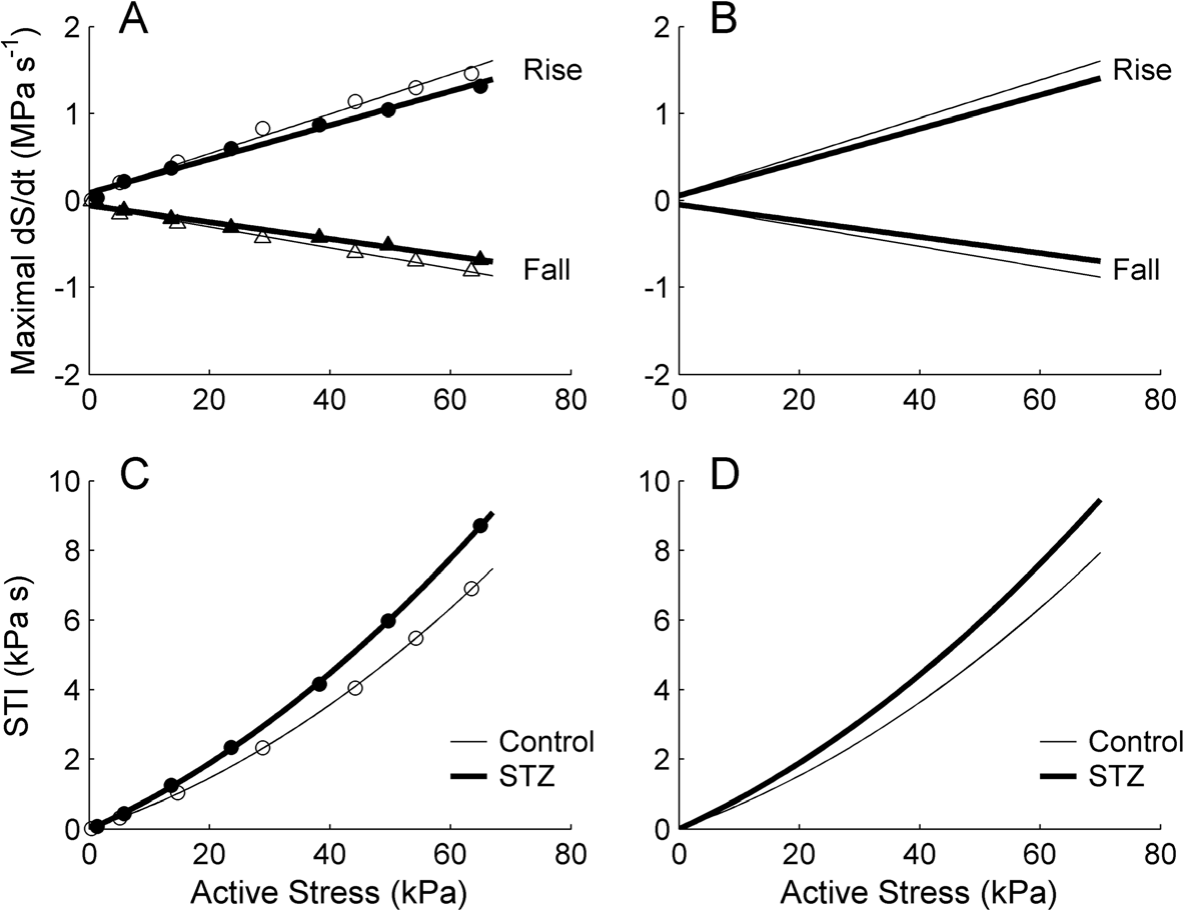
**Rates of rise and fall of isometric twitch stress, and stress-time integral, as functions of active stress, at 3 Hz stimulation.** Maximal rate of rise (upper lines) and rate of fall (lower lines) **(A)**, and stress-time integral (*STI*) **(C)**, as functions of active stress for a representative trabecula from the Control group (open symbols) and from the STZ group (filled symbols). Average relations **(B and D)** for the Control (thin lines) and the STZ (thick lines) trabeculae. The slopes of the relations shown in **B** and in **D** are significantly different between the Control and the STZ groups.

The maximal rates of rise (*+dS/dt*) and fall (−*dS/dt*) of isometric twitch stress were expressed as functions of active stress. The average relations describing *dS/dt* and active stress (Figure 4B) of the STZ groups were positioned lower than those of the Control groups, implying that diabetes slows the kinetics of isometric twitch stress. On the other hand, the average relation between stress-time integral (*STI*) and active stress (Figure 4D) was greater for the STZ trabeculae than for the Control trabeculae. This result reflects the fact that, at any given active stress, the STZ trabeculae have a greater value of *STI* due to their prolonged twitch duration (Figure 3), and their stress values were statistically not different to the Control values (Figure 2C).

### Isometric heat as functions of stress and stress-time integral

**Figure 5:**
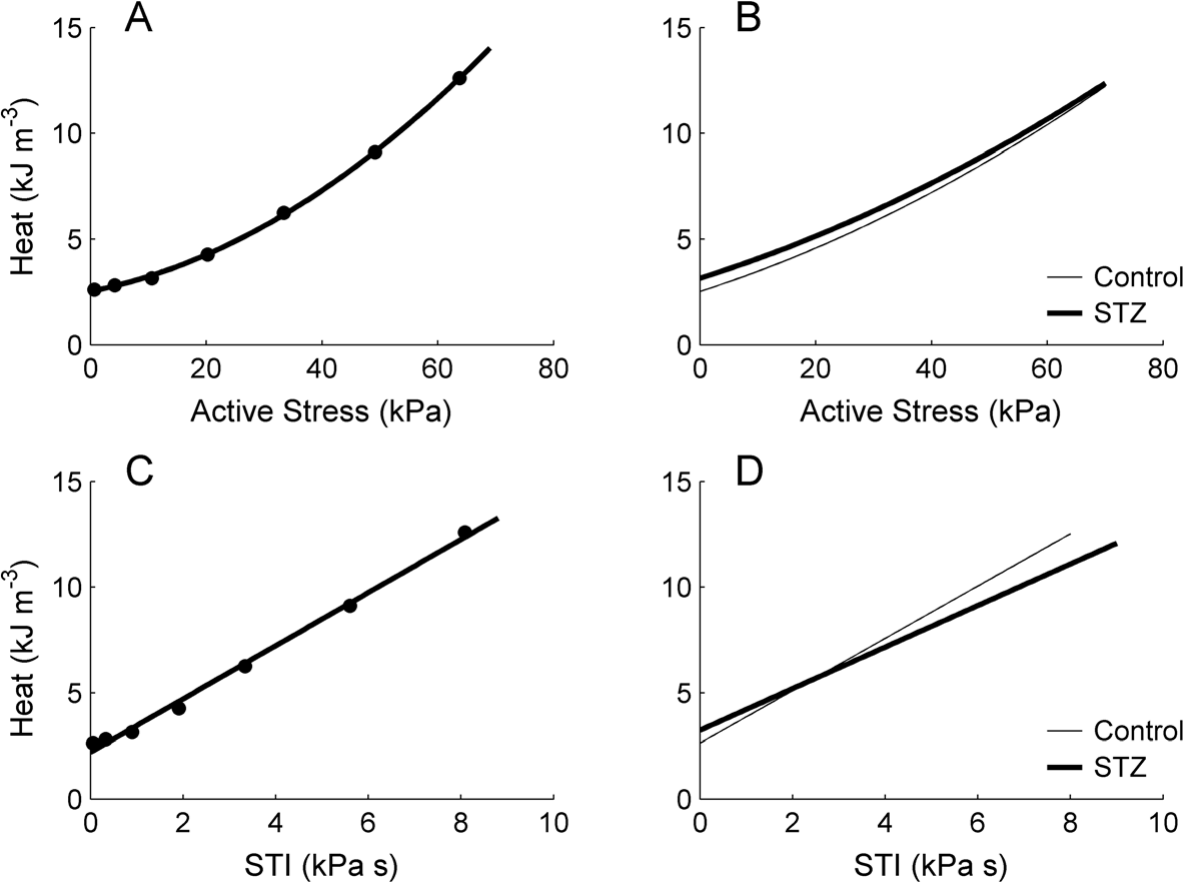
**Twitch heat as functions of active stress and stress-time integral at 3 Hz stimulation.** Representative **(A and C)** and average **(B and D)** heat-stress and heat-STI (stress-time integral) relations under isometric contractions at variable muscle lengths for Control (thin lines) and STZ (thick lines) trabeculae. The slope of the average heat-STI relation (in **D***)* is lower in the STZ group.

Rate of heat production (Figure 2B) was converted to heat per twitch by dividing by stimulus frequency (3 Hz). Steady-state values of twitch heat were plotted as functions of steady-state values of active stress (Figure 5A and B). Diabetes had no effect on the average heat-stress relation, or on the average heat at zero active stress (Control: 2.52 kJ m^-3^ ± 0.39 kJ m^-3^; STZ: 3.15 kJ m^-3^ ± 0.43 kJ m^-3^). Twitch heat was also plotted as a function of stress-time integral, *STI* (Figure 5C and D). Diabetes had no effect on the heat at zero active stress (Control: 2.63 kJ m^-3^ ± 0.38 kJ m^-3^; STZ: 3.24 kJ m^-3^ ± 0.42 kJ m^-3^), but lowered the slope (Control: 1.24 s ± 0.05 s; STZ: 0.98 s ± 0.05 s) of the average heat-STI relation. This is consistent with the diabetes-induced prolongation of twitch duration (Figure 3).

### Dynamic modulus

**Figure 6:**
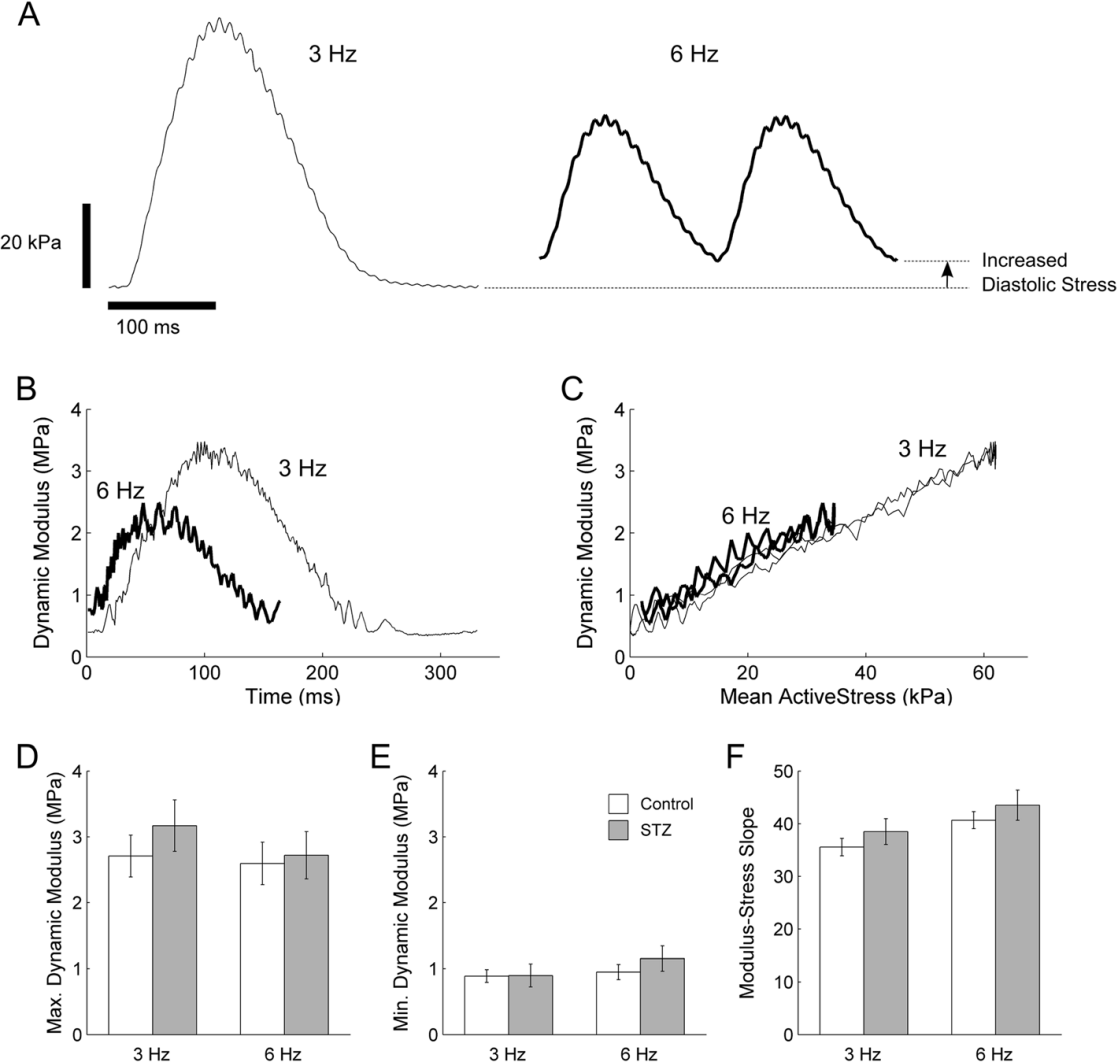
**Dynamic modulus at 3 Hz and at 6 Hz stimulations. (A)** Representative muscle length-perturbed twitch stresses at 3 Hz (thin trace) and 6 Hz (thick trace) stimulus frequencies. The arrow indicates the extent of increase of diastolic stress at 6 Hz. Representative calculated dynamic modulus throughout the time-course of a twitch **(B)** and as a function of mean active stress **(C)**. No significant difference in the average maximal **(D)** and average minimal **(E)** dynamic modulus, and the average slope of the modulus-stress relation (calculated by linear regression of the data in **C**) **(F)**, between the Control (open bars) and the STZ (filled bars) trabeculae.

Given the prolonged twitch duration (Figure 3), slowed kinetics and increased STI (Figure 4) of the diabetic trabeculae, we interrogated crossbridge status during the time-course of twitch stress production by measuring dynamic modulus. Dynamic modulus (dynamic stiffness normalised to muscle cross-sectional area and length) was calculated from the amplitudes of the sinusoidally-perturbed twitch stress (Figure 6A) and sinusoidally-perturbed muscle length (0.001 L_o_), and plotted against both the time-course (Figure 6B) and the amplitude (Figure 6C) of mean twitch stress. The maximal and minimal values of dynamic modulus, which occur respectively at the peak active stress and diastolic stress, were not different between the Control and the STZ trabeculae (Figure 6D and E). Likewise, the slopes of the modulus-stress relations (Figure 6C), calculated by linear regression analyses, were also not different between groups, at either 3 Hz or 6 Hz stimulus frequency.

### Twitch stress at 6 Hz stimulation

**Figure 7:**
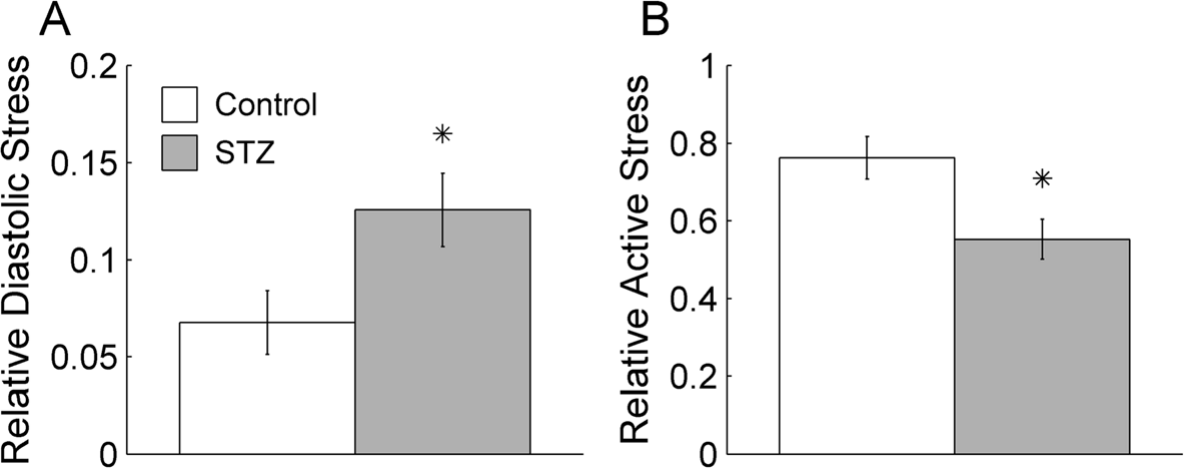
**Peak isometric twitch stress at 6 Hz stimulation.** Diastolic stress **(A)**, and active stress **(B)**, both expressed as fractions of the active stress at 3 Hz. *P < 0.05.

We reduced the diastolic period during a contraction by challenging the trabeculae to contract at a high stimulus frequency (6 Hz). At 6 Hz stimulation (and at *L*_*o*_), trabeculae experienced incomplete relaxation of stress between consecutive twitches, as shown by the double-head arrow in Figure 6A. We quantified this index by expressing its value as a fraction of the value of active stress at 3 Hz. As shown in Figure 7A, the average *relative* diastolic stress at 6 Hz was greater in the STZ trabeculae, indicating that the STZ trabeculae were unable to relax between twitches to the same degree as the Control trabeculae. Their average active stress at 6 Hz (as a fraction of that at 3 Hz) was lower than that of the Control trabeculae (Figure 7B). These results are consistent with the significantly prolonged twitch duration of the STZ trabeculae at 6 Hz. At 5% of peak active stress, their average contraction duration was 57.0 ms ± 1.2 ms (versus Control: 52.4 ms ± 1.2 ms), their average relaxation duration was 88.5 ms ± 1.1 ms (versus 84.2 ms ± 1.3 ms), and their average total duration was 145.5 ms ± 0.5 ms (versus 136.6 ms ± 1.9 ms). Likewise, at 50% of peak active stress, their average durations for contraction, relaxation, and total were, respectively, 37.7 ms ± 0.6 ms (versus 34.3 ms ± 0.9 ms), 47.0 ms ± 0.7 ms (versus 42.6 ms ± 1.4 ms), and 84.7 ms ± 0.8 ms (versus 77.0 ms ± 2.1 ms).

### Work-loop contractions

**Figure 8:**
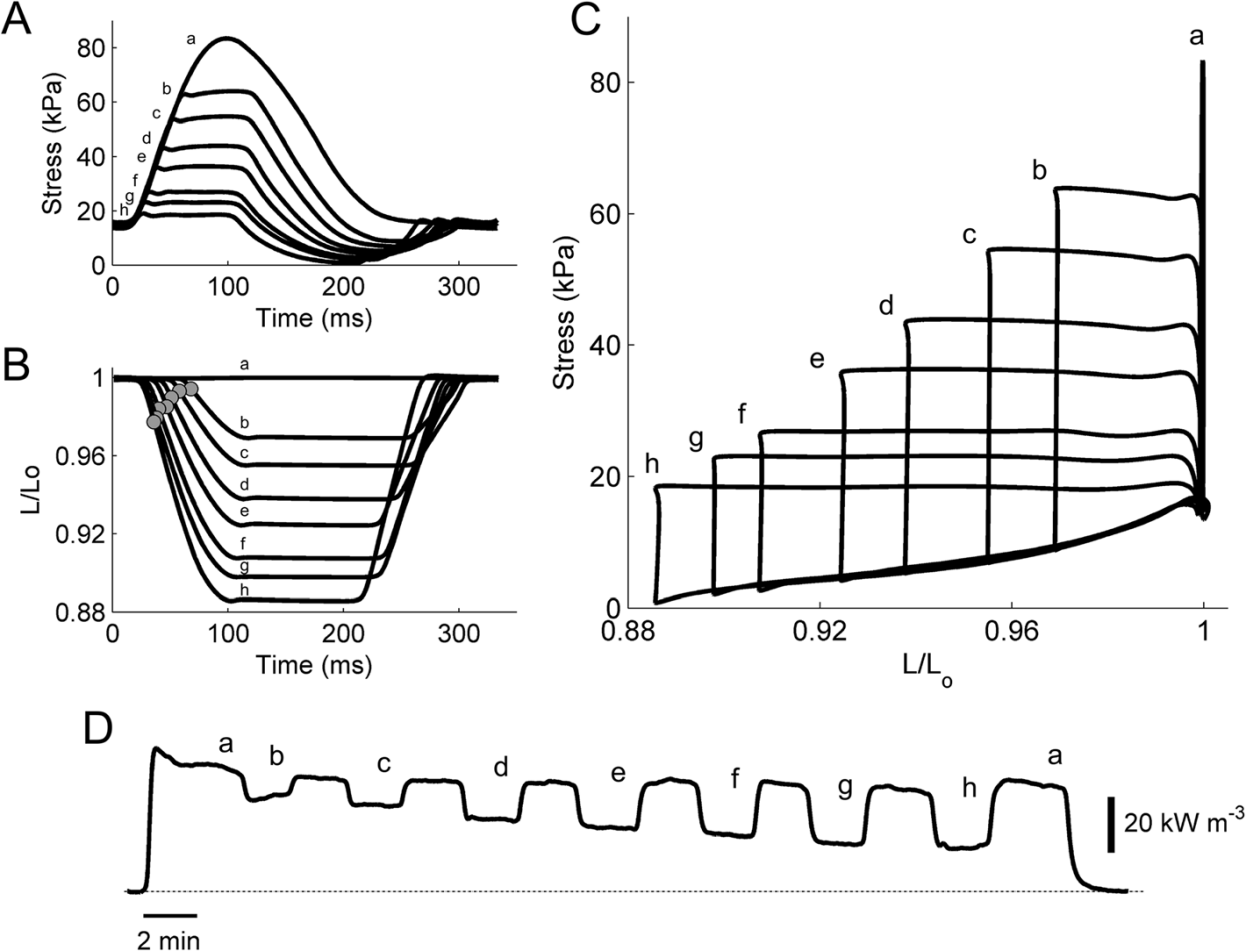
**Twitch stress, length change and rate of heat production of isotonic work-loops. (A)** Steady-state twitch stress of an isometric contraction (*a*) superimposed with work-loop twitch stress profiles at various afterloads (*b* – *h*) of a representative trabecula. **(B)** Corresponding length changes throughout the time-courses of twitch stresses in *A*. The grey circles indicate the locations at which velocities of muscle shortening were maximal. **(C)** Parametric plots of the data in *A* against those in *B*. **(D)** Record of rate of heat production for 7 variously-afterloaded work-loop contractions (*b* – *h*), bracketed by 8 isometric contractions (*a*).

Each trabecula was also subjected to work-loop contractions (at 3 Hz stimulus frequency) in the order of decreasing afterload. Each afterloaded work-loop contraction was interleaved with an isometric contraction. Figure 8A shows superimposed steady-state twitch stress for an isometric contraction (*a*) and for work-loop contractions (*b* - *h*). The associated change of muscle length is plotted in Figure 8B. Twitch stress is also plotted against muscle length (Figure 8C), and the resulting area of a work-loop stress-length relation quantifies the external work output of the trabecula. Rate of heat production accompanying the isometric and work-loop contractions during the entire period of experiment is depicted in Figure 8D.

### Duration of work-loop twitches

**Figure 9:**
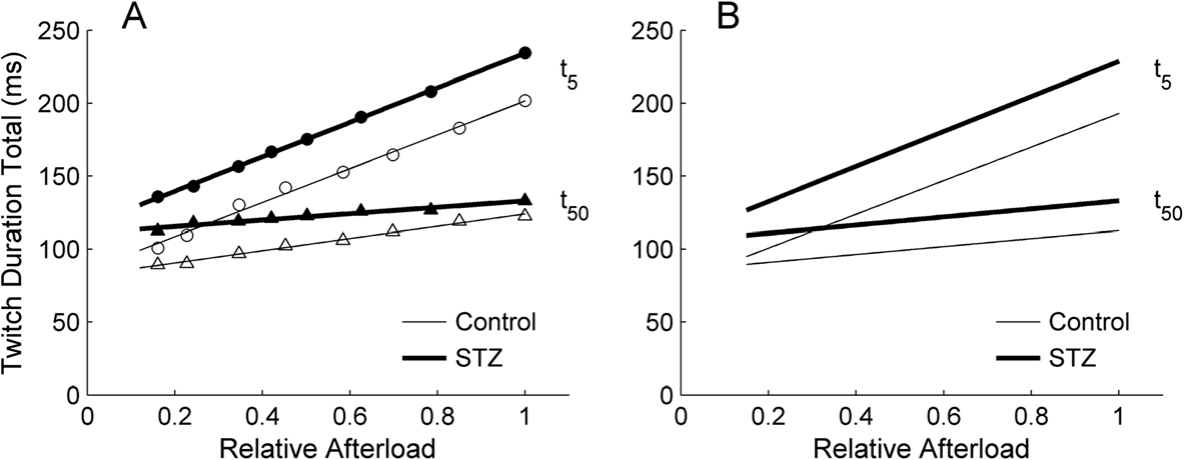
**Duration of work-loop twitches as functions of relative afterload. (A)** Total duration of work-loop twitch stress, quantified at 5% (upper lines) and 50% (lower lines) of afterload, for a representative trabecula from the Control group (open symbols) and from the STZ group (filled symbols). **(B)** Average relations for the Control (thin lines) and STZ (thick lines) trabeculae. No difference between average slopes of regression lines at either t_5_ of t_5o_ but their elevations higher in the STZ group.

The durations of the work-loop twitch profiles were quantified at 5% and 50% of peak stress, and plotted against afterload (relative to the isometric total stress), as shown in Figure 9. Diabetes prolonged the durations (5% and 50%) of work-loop twitches at all relative afterloads (Figure 9B).

### Extent of shortening, shortening velocity and shortening power

**Figure 10:**
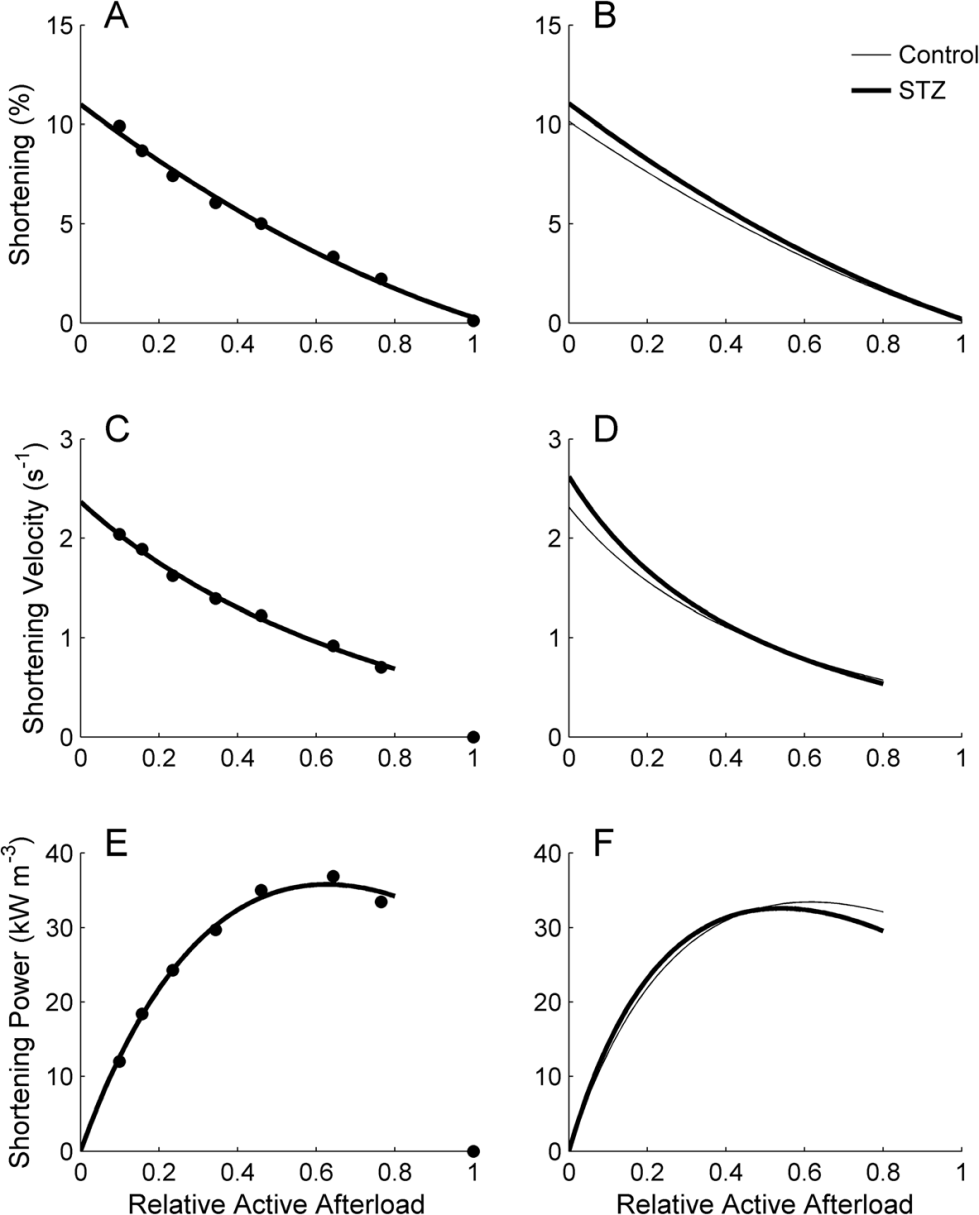
**Extent of shortening, shortening velocity and shortening power as functions of relative active afterload. (A and B)** Maximal extent of shortening (calculated from the end-systolic length in Figure 7C), **(C and D)** maximal velocity of shortening (at the times indicated by the circles in Figure 7B), and **(E and F)** maximal power of shortening (calculated as the product of velocity of shortening and active afterload) as functions of relative active afterload for a representative trabecula (left panels) and the average relations (right panels) for the Control (thin lines) and STZ (thick lines) trabeculae.

The extent of muscle shortening during work-loop contractions was calculated from the end-systolic length (at which point the muscle transitions from the isotonic shortening phase to the isometric relaxation phase of the work-loop; Figure 8A-C), and expressed as a percentage of *L*_*o*_. Diabetes had no effect on the average peak extent of shortening (extrapolated to zero relative active afterload); Control: 10.2% ± 0.5%, STZ: 11.0% ± 0.8% (Figure 10B). Shortening velocity was calculated as the maximal slope of the length-time trace (Figure 8B, grey circles) and normalised to *L*_*o*_. Diabetes had no effect on the average peak shortening velocity; Control: 2.27 s^-1^ ± 0.15 s^-1^; STZ: 2.72 s^-1^ ± 0.27 s^-1^ (Figure 10D). Shortening power was computed as the product of shortening velocity and active afterload. Once again, diabetes had no effect on this index of mechanics, Control: 35.6 kW m^-3^ ± 5.2 kW m^-3^; STZ: 32.4 kW m^-3^ ± 3.8 kW m^-3^ (Figure 10F), quantified at a *relative* active afterload of 0.6 (i.e., in the vicinity of peak shortening power).

### Heat, change of enthalpy, work and efficiency

**Figure 11:**
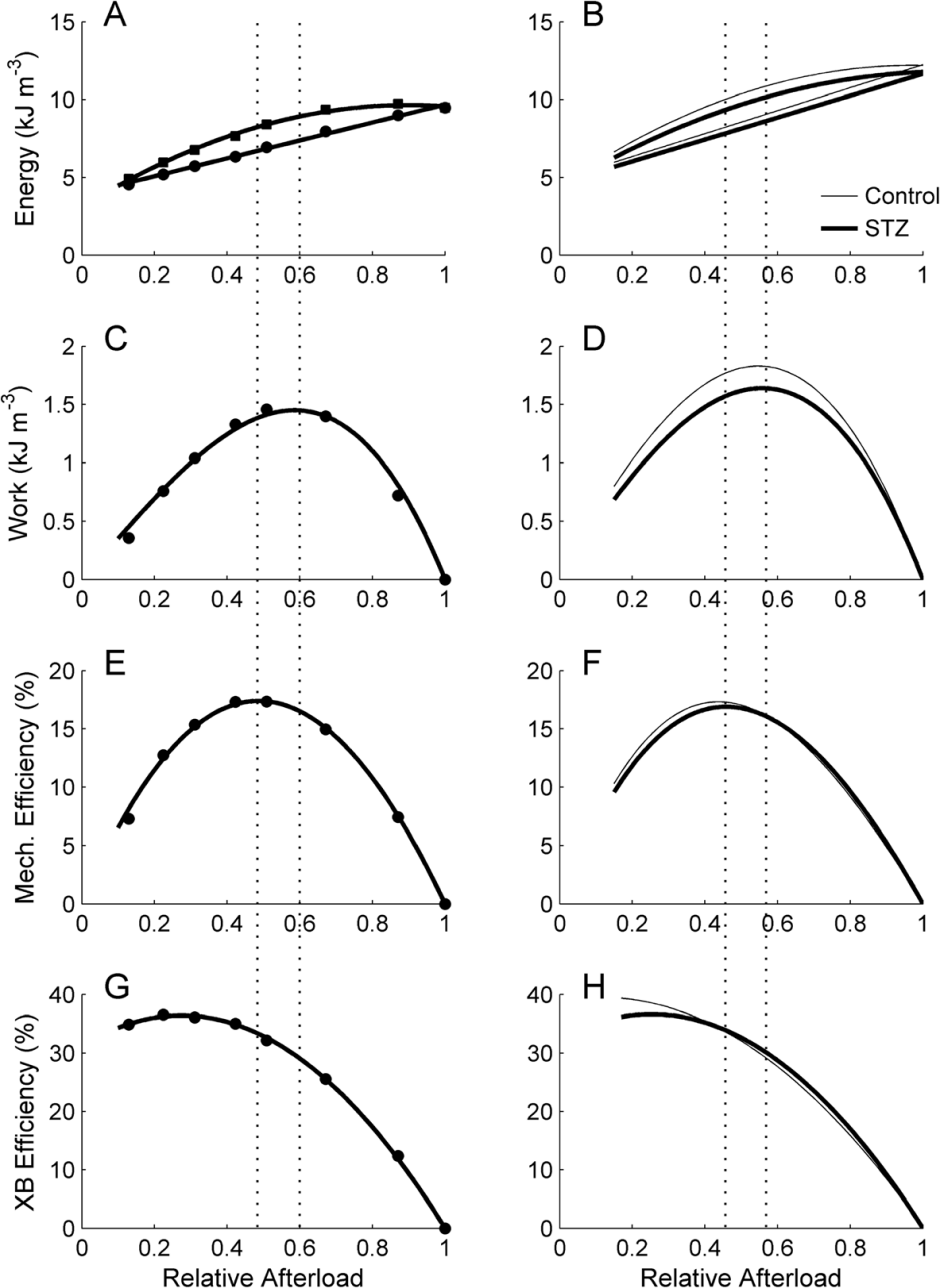
**Energy expenditure, work output and efficiency as functions of relative afterload. (A)** Change of enthalpy (squares) and heat (circles), **(C)** work output, **(E)** mechanical efficiency and **(G)** crossbridge (‘XB’) efficiency as functions of relative afterload for a representative trabecula. The corresponding average relations for the Control (thin lines) and STZ (thick lines) trabeculae are shown in **B**, **D**, **F** and **H**, respectively. The left and the right vertical dotted lines indicate, respectively, the relative afterloads for peak mechanical efficiency and for peak work.

We plot the heat production, external work output, change of enthalpy (work plus heat), mechanical efficiency (the ratio of work to change of enthalpy) and crossbridge efficiency as functions of relative afterload in Figure 11. Crossbridge efficiency was revealed by subtracting, from the denominator of the expression for mechanical efficiency, the heat value at zero relative afterload, extrapolated using the linear heat-afterload relation (Figure 11A and B). The average extrapolated heat values did not differ between the Control and the STZ trabeculae (4.87 kJ m^-3^ ± 0.62 kJ m^-3^ versus 4.63 kJ m^-3^ ± 0.69 kJ m^-3^).

We interpolated the values of the above variables at two values of relative afterload: one when mechanical efficiency was at its peak (0.44 S/S_o_ - 0.47 S/S_o_) and the other when work was at its peak (0.53 S/S_o_ - 0.56 S/S_o_). Peak work (1.78 kJ m^-3^ ± 0.29 kJ m^-3^ versus 1.61 kJ m^-3^ ± 0.20 kJ m^-3^) and peak mechanical efficiency (17.5% ± 1.0% versus 17.4% ± 1.3%) did not differ between the Control and the STZ trabeculae. At the relative afterload that gave peak mechanical efficiency, the heat (8.11 kJ m^-3^ ± 0.95 kJ m^-3^ versus 7.84 kJ m^-3^ ± 0.84 kJ m^-3^), change of enthalpy (9.86 kJ m^-3^ ± 1.23 kJ m^-3^ versus 9.34 kJ m^-3^ ± 0.99 kJ m^-3^), work (1.71 kJ m^-3^ ± 0.28 kJ m^-3^ versus 1.55 kJ m^-3^ ± 0.19 kJ m^-3^), and crossbridge efficiency (34.6% ± 1.3% versus 33.8% ± 1.5%) were not different between Control and STZ trabeculae. Likewise, at the relative afterload at which work peaked, the heat (8.82 kJ m^-3^ ± 1.03 kJ m^-3^ versus 8.52 kJ m^-3^ ± 0.93 kJ m^-3^), change of enthalpy (10.64 kJ m^-3^ ± 1.34 kJ m^-3^ versus 10.06 kJ m^-3^ ± 1.11 kJ m^-3^), mechanical efficiency (16.8% ± 0.9% versus 16.7% ± 1.1%) and crossbridge efficiency (31.3% ± 1.3% versus 30.9% ± 1.5%) were not different between groups.

## Discussion

In this study, we present the first results of STZ-induced diabetes on the energetics of isolated left-ventricular (LV) trabeculae, acknowledging that diabetes prolongs twitch duration. We measured mechanical work output and heat production. Several additional first results from diabetic cardiac preparations are also presented: (1) twitch duration as a function of stress or afterload, (2) heat-stress and heat-STI (stress-time integral) relations obtained under isometric contractions, (3) dynamic modulus as a function of isometric twitch stress, and (4) heat-afterload, work-afterload and efficiency-afterload relations derived from work-loop contractions. Given these results, we are in a position to reconcile the performance of the isolated cardiac muscle with that of the intact heart.

### Relation of trabecula performance to that of the whole heart

Diabetes prolongs twitch duration in both isolated trabeculae and the heart, but contractile dysfunction prevails only in the heart. In our recent study of isolated, working, hearts [50], we showed that the maximal afterload achievable by the diabetic heart (i.e., when its aortic outflow is near zero) is substantially lower than that of Control hearts. Thus, at high afterloads, the work output of the diabetic heart is compromised. In contrast, diabetes does not affect the stress development of isolated trabeculae (Figure 2C) nor any other index of mechano-energetic performance, with the single exception of twitch duration, which is prolonged in diabetic preparations under both isometric (Figure 3) and work-loop (Figure 9) protocols. Because of the comparability of mechanical performance, the efficiency-afterload curve of the diabetic trabeculae is the same as that of the Control group (Figure 11F). Note that the null effect of diabetes on the *absolute* stress production of trabeculae allows us to express efficiency as a function of *relative* afterload.

When a trabecula is isolated from a diabetic heart, it is freed from the effects of insufficient ventricular filling. It can now be stretched to *L*_*o*_, where its stress development is found to be as high as that of healthy Control trabeculae (Figure 2C). This finding gives us confidence that sub-optimal ventricular filling, as a consequence of prolonged twitch duration and attendant abbreviation of diastolic interval, is sufficient to explain LV contractile dysfunction in the heart. Hence, twitch prolongation alone is sufficient to develop LV contractile dysfunction at high afterloads. That is, prolonged twitch duration reduces the period, and hence the extent, of diastolic refilling, leading to failure to generate the high pressures required to overcome high afterloads. This, however, does not compromise peak stress development at the level of isolated myocardial tissue. Consequently, diabetes does not affect the efficiency-afterload relation of isolated trabeculae, as hypothesised.

To explain further the apparent contradiction between the contractile performance of the heart and isolated trabeculae, it is necessary to consider four facts: (1) twitch duration increases with increasing muscle length (Figure 3), as well as with afterload (Figure 9), (2) for a given pacing rate, prolongation of twitch duration is associated with a reduction of the diastolic period (Figure 2A inset and Figure 8A), i.e., reducing the time for diastolic filling of the ventricles or re-lengthening of isolated trabeculae, (3) the extent of reduction of the diastolic period increases with increasing stimulus frequency (Figure 6A) or heart rate, and (4) the reduced intrinsic heart rate of the diabetic animal [6,32,40,42,44,50].

Consideration of these facts requires comparison of twitch duration between the diabetic heart and the Control heart at the same rate of stimulation. This can be achieved by externally pacing the heart. In our heart study, we paced the hearts to beat at 4 Hz, which is above their intrinsic rate at 32°C: 2.6 Hz and 2.9 Hz for the STZ and Control groups, respectively [50]. We did not measure twitch duration of the heart and are unaware of any study that has paced the diabetic heart at the same rate as the Control heart in order to quantify the diabetes-induced prolongation of twitch duration. However, we can make use of the trabecula results of the current study in order to draw an inference concerning the contractile dysfunction of the diabetic heart at high afterloads. We electrically stimulated diabetic trabeculae to contract at the same rate as their controls (3 Hz). By doing so, we saw a diabetes-induced prolongation of twitch duration (Figure 3), consistent with the results of others in isolated papillary muscles [2,3,28,30,34-37], ventricular trabeculae [6,12], and single myocytes [13,31,38,39]. We infer that the same behaviour occurs in the diabetic heart.

Collectively, our results allow us to infer that the diastolic filling time of the diabetic heart is reduced, and is disproportionately reduced as the afterload challenge is increased. Hence, at sufficiently high afterloads, the diabetic heart suffers inadequate ventricular filling and, consequently, reduced aortic outflow. The healthy heart, in contrast, can pump to a higher afterload given its longer period of diastolic filling.

### Frequency-dependence of peak active stress

The null effect of diabetes on peak active stress of isolated trabeculae renders our hypothesis valid. Many previous studies have also reported a null effect of diabetes on peak active stress (i.e., when the muscle is held at *L*_*o*_) at stimulus frequencies ≤ 1 Hz in isolated papillary muscles [2,3,28,30,34-37] and in isolated single myocytes [46,47,49]. But, there are several studies showing lower contractility of diabetic myocytes at stimulus frequencies ≤ 1 Hz [31,38,57] as well as at 2 Hz [29]. The reason for these discrepant literature findings is unclear given that these experiments were performed at comparable temperatures (30°C – 37°C). Given these ambiguous literature reports, we compared the results of four independent studies which examined the effect of stimulus frequency at a fixed temperature: Cameron et al. [34] and Nobe et al. [3] on isolated papillary muscles, Zhang et al. [6] on isolated LV trabeculae, and Ren and Davidoff [39] on isolated single myocytes. The first study showed no effect of diabetes on peak active stress at 30°C and at a range of stimulus frequency between 0.1 Hz and 4 Hz. The second reported no effect of diabetes on peak active stress at 36°C between 0.2 Hz and 5 Hz. The third study, at 37°C, showed no effect of diabetes at 1 Hz or 2.5 Hz, but lower values at 5 Hz and 7 Hz. Lastly, the fourth study, also at 37°C, found the differential effect of diabetes to disappear at 5 Hz, but not at frequencies below 2 Hz. We have no explanation for the discrepant findings between the latter studies. We conclude that the effect of diabetes on peak active stress production appears to be dependent on stimulus frequency. Our results, at 32°C, show no effect of diabetes on the peak active stress production at 3 Hz stimulation, but a negative effect at 6 Hz (Figure 7B).

### Frequency-dependence of diastolic stress

At 6 Hz, relaxation of the twitch is incomplete in both Control and diabetic trabeculae (Figure 6A), resulting in the elevation of diastolic stress between successive twitches. We have previously shown [53] that, in healthy trabeculae, incomplete relaxation is initiated by elevation of diastolic intracellular Ca^2+^ and a subsequent decreased myofilament sensitivity to Ca^2+^, and is not due to inadequate supply of either glucose or oxygen. Compared with the Control trabeculae, the diabetic trabeculae experience greater diastolic stress (Figure 7A), indicating that they fail to relax between twitches as fully as that of the Control. The inability of the diabetic trabeculae to achieve complete relaxation at 6 Hz is exacerbated by their prolonged twitch duration. The diabetic trabeculae have less time to relax before the next twitch commences, and consequently, they experience a greater extent of incomplete twitch relaxation. This result implies that the diastolic intracellular Ca^2+^ at 6 Hz is greater in the diabetic trabeculae, and the decrease of sensitivity of myofilaments to Ca^2+^ is more severe in the diabetic trabeculae. The latter implication is consistent with the findings of Zhang et al. [12] and Op Den Buijs et al. [58] showing a diabetes-induced decrease of Ca^2+^ responsiveness.

### Dynamic stiffness as a probe of cross-bridge function

Because of the well-documented negative effect of diabetes on actomyosin ATPase activity (see above), we invoked the technique of high-frequency, low-amplitude oscillation of muscle length in order to interrogate crossbridge function. We measured dynamic modulus (dynamic stiffness normalised to muscle dimensions) to quantify the status of crossbridges (i.e., the number attached and their individual stiffness) throughout the time course of twitch stress production.

We find no effect of diabetes on dynamic modulus (Figure 6). The linear relation between dynamic modulus and active stress (which implies that the net number of attached crossbridges changes linearly with stress production) is unaffected by diabetes. The maximal and minimal values of dynamic modulus, obtained at the peak stress and diastolic stress, respectively, are also unaffected by diabetes. Our modulus result, at high perturbation frequency (100 Hz), is consistent with that of Metzger et al. [59], who demonstrated that the modulus-tension relations are similar between control and β-MHC-expressing ventricular myocytes of the hypothyroid rat. These authors inferred that “… force production per strong crossbridge interaction, or the distribution of force-generating crossbridge states, is not cardiac MHC isoform dependent”. Thus, our results suggest that, although diabetes prolongs twitch duration, it does not affect the net number of crossbridges attached or their individual stiffness. This inference further suggests that the contractile dysfunction at the whole heart level is predominantly due to insufficient LV diastolic filling, and not to crossbridges status *per se*.

### Heat production

During a train of isometric contractions, trabeculae perform negligible external work, and hence the metabolic change of enthalpy (heat plus work) consists almost entirely of heat. We plotted isometric heat as functions of both developed stress and *STI* (Figure 5). Both relations have previously been used for studying the mechano-energetic of healthy, non-diabetic, isolated papillary muscles [60,61] and trabeculae [53,62] under a variety of experimental conditions. In the present study, we observed the heat-stress relation to be slightly curvilinear but the heat-STI relation to be linear. The extrapolated y-intercepts of these relations, which did not differ and were unaffected by diabetes, are presumed to estimate ‘activation heat’, i.e., the energy expenditure associated with Ca^2+^ cycling by the sarcoplasmic reticulum Ca^2+^-ATPase and Na^+^ extrusion by the sarcolemmal Na^+^-K^+^ ATPase. Note that the null effect of diabetes on activation heat obtained in this study is *not* at odds with reports showing decreased activities of both the sarcoplasmic reticular Ca^2+^-ATPase [17-22] and the sarcolemmal Na^+^-K^+^-ATPase [14-16] in diabetic cardiac preparations. This is because a decreased activity of an ATPase does not imply decreased metabolic energy expenditure, since the same total amount of heat could be produced independent of the *rate* of ATP hydrolysis.

Under the assumption that the activation heat is independent of developed stress, the monotonic increase of heat output with increasing stress reflects the metabolic energy expenditure of the contractile apparatus by the actin-activated myosin-ATPase. The inverse of the slope of the heat-stress relation (Figure 5B) is hence an index of crossbridge economy. The absence of a difference in the magnitudes of this index between the healthy and STZ-treated trabeculae implies that diabetes does not render the contractile apparatus less ‘economic’, despite reports showing reduced *rate* of ATP hydrolysis by myofibrillar-ATPases [23-28]. Since twitch duration is prolonged but twitch stress is unaffected, the area under the time-course of the twitch (i.e., its stress-time integral, *STI*) is increased in the diabetic preparations (Figure 4D). For a given value of active stress, heat production is unchanged in the diabetic preparations (Figure 5B). Given the effects of diabetes on twitch duration, twitch stress and twitch heat, the slope of the heat-STI relation is destined to be lower in the intact diabetic trabeculae, as confirmed in Figure 5D.

Our results, showing the effect of diabetes on the heat-stress relation of *intact* LV trabeculae at 32°C, are not comparable with those of Rundell et al. [63] who used *skinned* RV trabeculae at 20°C. Those authors reported decreased tension cost (indexed by the slope of the linear ATPase-tension relation) in the diabetic group, which they attributed to reduced expression of the α-MHC (fast) isoform. Studies by Holubarsch et al. [64,65], using *intact* LV papillary muscles at 21°C, showed that hypothyroid rats (expressing β-MHC) have a lower slope of the heat-STI relation, in agreement with our result (Figure 5D). Thus, the use of intact versus skinned preparations may be responsible for the inconsistency of our heat-stress relation with that of the ATPase-tension relation of Rundell et al. [63].

### Null effect of diabetes on muscle shortening

The heart does not ever perform purely isovolumic contractions. Rather, it reduces volume in the process of ejecting blood. During the ejection period, when the outflow valves are open, pressure and volume change continuously. We approximated the pressure-volume loops of the heart by subjecting each trabecula to a series of isotonic stress-length loops (Figure 8). Using the data obtained during the isotonic shortening phases of the stress-length loops, we quantified three parameters related to muscle shortening: (i) the peak velocity of shortening, computed as the maximal slope of the length-time trace during the isotonic shortening phase of the work-loop (Figure 8B), (ii) the power of shortening, which is the product of maximal velocity of shortening and active afterload, and (iii) the peak extent of shortening, calculated as the relative length at which the trabecula transitioned from isotonic shortening to isometric relaxation (Figure 8B and C). The latter (end-systolic) length corresponds to the end-systolic volume of the heart. We found that the peak extent of shortening, as well as its peak velocity (extrapolated to the y-intercept), was comparable between control and diabetic trabeculae (Figure 10B and D). Shortening power also did not differ between the two groups (Figure 10F). These results imply that shortening is unaffected by diabetes, and are thus in accord with the findings of many [13,46-49] but not all [31,38,57] single-myocyte studies.

Our finding of an absence of effect of diabetes on shortening velocity is not consistent with those studies showing a shift of myosin heavy-chain expression from the α to the β isoform [20,24,26,29,30]. We note that the velocity of shortening of diabetic preparations is dependent on extracellular Ca^2+^ concentration. Fein et al. [36] found that muscle shortening, both its velocity and its extent, were similar between control and diabetic rat papillary muscles at a bath Ca^2+^ concentration of 0.6 mM. But at an elevated (and non-physiological) Ca^2+^ concentration (2.4 mM), the shortening velocities of the diabetic preparations were lower than those of the control group. Similarly, Siri et al. [66] showed the peak extent of shortening to be unchanged, but shortening velocity reduced, in diabetic-hypertensive rat papillary muscles tested with 2.4 mM Ca^2+^. Joseph et al. [67] also reported decreased peak shortening velocity in the diabetic papillary muscle at 2.5 mM Ca^2+^. Whether the activity of the MHC is Ca^2+^-dependent requires future experiments. Our results, at physiological Ca^2+^ (1.5 mM), show no effect of diabetes on muscle shortening.

### Crossbridge efficiency

Lastly, crossbridge efficiency, revealed by subtracting activation heat (extrapolated from the heat-relative afterload relation shown in Figure 11B) from the denominator of the expression for mechanical efficiency, is also indifferent to diabetic status (Figure 11H). The null effect of diabetes on crossbridge efficiency is consistent with that reported by Joseph et al. [67]. These authors *calculated*, using their experimental force and velocity data, together with several assumptions about crossbridge characteristics and energetics, a value of crossbridge efficiency of 30%, in agreement with our experimentally measured values (Figure 11H).

## Conclusions

Our collective results from isolated LV trabeculae allow us to infer that diabetes-induced prolongation of the twitch reduces the period, and hence the extent, of left-ventricular diastolic filling. In consequence, the diabetic heart is incapable of pumping at afterloads exceeding about two-thirds of the maximum achievable by the healthy heart [50], resulting in a left-shift of its efficiency-afterload curve. However, in isolated trabeculae, which are freed from the complication of insufficient diastolic filling, the efficiency-afterload curve is unaffected by diabetes. We conclude that the peak efficiency of the heart and its tissues is unaffected by diabetes.

## Abbreviations

STZ: Streptozotocin; LV: Left ventricle or left-ventricular; STI: Stress-time integral; ATP: Adenosine triphosphate; MHC: Myosin heavy chain.

## Competing interest

The authors declare that they have no competing interests.

## Authors’ contributions

J-CH conceived and designed the study, performed experiments and was responsible for acquisition, interpretation, analysis and statistical analysis of data and drafting the manuscript. DL participated in study concept and design and contributed to acquisition, interpretation and statistical analysis of data and drafting of the manuscript. AT participated in study design, contributed to acquisition and interpretation of data. KT participated in analysis and interpretation of data and drafting of the manuscript. PN participated in interpretation of data. All authors participated in critical discussion and approved the final version of the manuscript.
